# The speciation of the proteome

**DOI:** 10.1186/1752-153X-2-16

**Published:** 2008-07-18

**Authors:** Peter R Jungblut, Hermann G Holzhütter, Rolf Apweiler, Hartmut Schlüter

**Affiliations:** 1 Max Planck Institute for Infection Biology, Core Facility Protein Analysis, Berlin, Germany; 2 European Bioinformatics Institute, Cambridge CB10 1SD, UK; 3 Charité Berlin, Institut für Biochemie, Berlin, Germany

## Abstract

**Introduction:**

In proteomics a paradox situation developed in the last years. At one side it is basic knowledge that proteins are post-translationally modified and occur in different isoforms. At the other side the protein expression concept disclaims post-translational modifications by connecting protein names directly with function.

**Discussion:**

Optimal proteome coverage is today reached by bottom-up liquid chromatography/mass spectrometry. But quantification at the peptide level in shotgun or bottom-up approaches by liquid chromatography and mass spectrometry is completely ignoring that a special peptide may exist in an unmodified form and in several-fold modified forms. The acceptance of the protein species concept is a basic prerequisite for meaningful quantitative analyses in functional proteomics. In discovery approaches only top-down analyses, separating the protein species before digestion, identification and quantification by two-dimensional gel electrophoresis or protein liquid chromatography, allow the correlation between changes of a biological situation and function.

**Conclusion:**

To obtain biological relevant information kinetics and systems biology have to be performed at the protein species level, which is the major challenge in proteomics today.

## Introduction

Many paradigm changes were caused by speciation. The speciation of all materials in chemical elements and the establishment of the periodic system by Mendeleev in 1869 was the beginning of chemical science and modern chemical industry. The ground-breaking work of Carl von Linné, who developed the *Systemae Naturae *in 1735 [1], was the basis for the theory of evolution by Charles Robert Darwin in his famous book "*On the Origin of Species by Means of Natural Selection, or The Preservation of Favoured Races in the Struggle for Life*" in 1859 [2] and a big step forward for modern medicine. Today another step forward in life sciences could be the speciation of the proteins into protein species and the acceptance of the protein species as the functional unit.

The vast amount of genomic, transcriptomic and proteomic data now at our fingertips enables us to recognize the tremendous diversity of proteins as distinguishable protein species with different structures and functions. In the 19^th ^century proteins were anticipated as amorphous mass. Only at the beginning of the 20^th ^century did it become clear that the proteins may be distinguished in different individual forms. The amino acids were recognized as the building blocks of proteins and their composition as a characteristic of a certain protein. Therefore, a differentiation became possible by the different content of different amino acids. Chromatography and differential solubility was used to separate and purify proteins with different function. Later it became obvious that the amino acid sequence is important for the definition of the function. Proteins can be distinguished according to their structure and function. In economics the process of the occurrence of new modified forms of one product is termed diversification; in life sciences the same process should be named speciation. The names used to identify proteins show that different polypeptides were named according to the description of their function.

The deciphering of the genetic code revealed that the amino acid sequence is determined in the DNA, and that the amino acid sequence reflects genomic information. Genetics culminated in the deciphering first of the genomes of microorganisms, and in 2002 of the human genome, with predictions of thousands of proteins, from which only a small proportion are experimentally accessible to date. The elucidation of complete DNA sequences and prediction of genes of *Haemophilus influenzae *[3] and human [4] were landmarks enabling detailed proteomic identification by mass spectrometry. In July 2008 there are 727 microbial genomes sequenced and 1108 are in progress [5]. For eukaryotes there are 23 completed genomes and 474 are in preparation [6].

In microorganisms with small genomes such as *Mycoplasma pneumoniae *(573 ORFs) proteome coverage of up to 80% [7] was reported. For bacteria with larger genomes (2000 to 4000 ORFs) it can be expected that a sample from one biological situation, prepared and analysed by one procedure, will give access to about 10–50% of the proteins predicted by their genome [8,9]. In human pancreatic cells 3365 proteins [10] and in mouse brain 7792 proteins, covering about 34% of the predicted mouse proteome [11] were identified. The use of the gathered knowledge presumes optimal data storage and data mining tools, for which a well defined terminology is a prerequisite.

Here we present a critical view on the terminology in proteomics and define the protein species chemically as the smallest unit, which can be correlated to a function. Only with a precise terminology we will be successful in theoretical proteomics and systems biology.

### Proteome definition

Improvements in chromatographic and electrophoretic methods resulted as early as 1970 in the separation and characterization of about 60 ribosomal proteins [12]. The combination of two high-resolution methods, isoelectric focusing and SDS polyacrylamide gel electrophoresis allowed the separation of several hundred proteins of a complete organism, the bacterium *Escherichia coli *[13]. The proteins were separated in a gel and appeared as spots in a two-dimensional pattern. By increasing the size of these gels up to 30 × 40 cm, the resolution was increased to more than 10000 spots per gel [14]. This technique, combined with methods for identification of proteins from gels, such as N-terminal sequencing [15,16] and mass spectrometry with soft ionization procedures [17-19], made it possible to perform protein analysis at a genomics scale [20]. This was the beginning of a new scientific discipline: Proteomics. The proteome has been defined as the **pro**tein complement of the gen**ome **[21]. Proteomics is the systematic study of the many and diverse properties of proteins in a parallel manner, with the aim of providing detailed descriptions of the structure, function and control of biological systems in health and disease [22].

### Gene expression and protein expression – a clear definition and a fuzzy term

**Figure 1:**
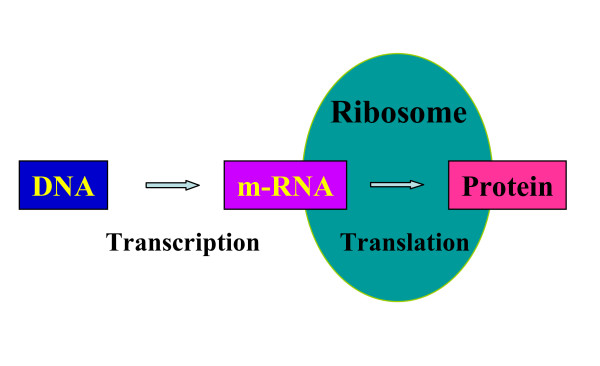
The central biochemical dogma.

**Figure 2:**
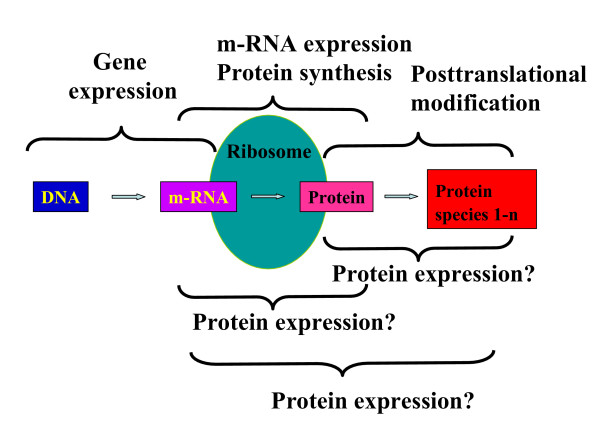
The attempt to define the term protein expression within an expanded model of the central biochemical dogma. At present the term protein expression is not clearly defined. It is used in the literature for different steps between RNA and protein species.

The connection between genes and proteins is described by the central dogma (Figure 1). The genes are expressed and during this process the genetic information is transcribed from DNA, to RNA and then translated from RNA to proteins. In analogy to the term gene expression the term 'protein expression' was introduced by Wilkins and Gooley, 1997 [23] into the proteome definition: "In proteome projects, which aim to identify and characterize all proteins expressed by an organism or tissue, the identification of proteins is central". Semantically the process of protein expression should start with a protein and result in a modified protein (Figure 2). The term protein expression is however very rarely used in this sense, but tends rather to be used in the context of translation or protein synthesis, which is semantically not correct and further leads to misunderstandings when attempts are made to measure protein expression by two-dimensional gel electrophoresis or liquid chromatography.

**Figure 3:**
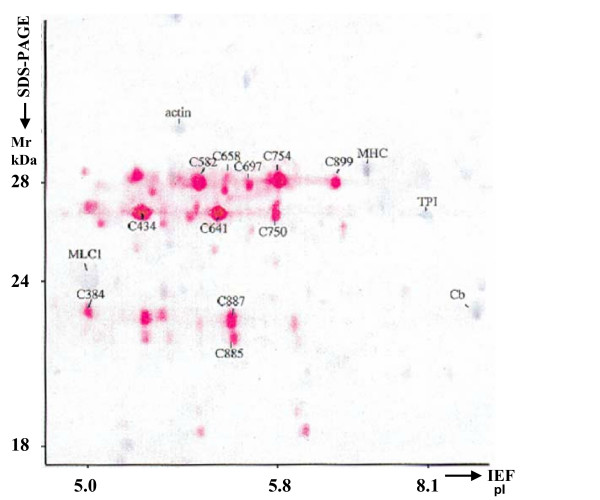
Part of a human myocardial 2-DE pattern. The proteins were blotted onto a PVDF membrane, immunostained with an antibody against Hsp27 (red spots), and counterstained with Coomassie Brilliant Blue R250 (blue spots) [24]. Spot numbers refer to the human heart high-performance 2-DE database [51]. The spot labelled with actin represents a fragment of actin. MLC1, myosin light chain 1, MHC, myosin heavy chain fragment, TPI, triosephosphate dehydrogenase, Cb, alpha-crystallin b chain.

The misleading character of this terminology is outlined in the example of a 2-DE analysis of heat-shock protein 27 (Hsp27) from human heart [24] (Figure 3). Hsp27 occurs in 59 spots of this 2-DE pattern. A different position in a 2-DE gel must be the result of a different chemical structure of the protein. It becomes obvious that a single spot cannot represent protein expression in the sense of protein synthesis, because protein synthesis also leads to at least 58 other modified forms of the protein. Attempting to account for all forms of the protein by adding up the intensities of the 59 spots does not really help to solve the problem, because it cannot be guaranteed that other forms of the protein exist, which were not resolved on the 2-DE gel. Additionally and even more importantly, is the fact that it cannot be decided whether the synthesis of the many protein forms was an earlier process or actually caused by the biological effect currently under investigation, or indeed whether the direct synthesis product was degraded or transported out of the cell or even the tissue.

In light of these considerations, it has to be accepted that all that can be measured on a 2-DE gel or in an LC run, is the amount of a protein (LC) or more precisely the amount of a particular form of a protein (2-DE) in a specific biological situation and experimental setting. The particular form of a protein we observe may result from transcripts from indistinguishable genes, indistinguishable parts of genes or from post-translational modifications.

### Protein species – the smallest unit of the proteome

**Figure 4:**
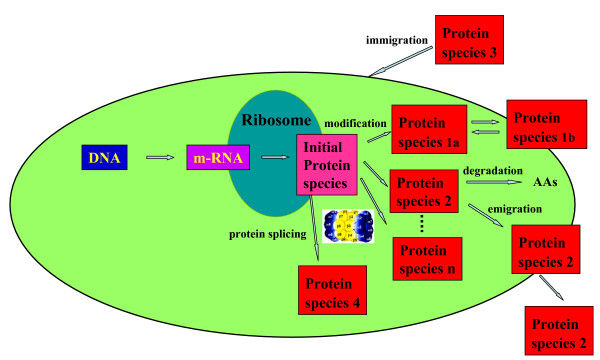
Extended central biochemical dogma: potential modes of speciation of a protein.

As outlined above, the protein expression concept is misleading and ignores the formation of different forms of proteins, each of which may have specific functions. These forms represent a new level of speciation: A protein derived from a given transcript diversifies into different protein species. The term protein species was defined by its chemical structure by Jungblut et al, in 1996 [25] and defines the smallest unit of the proteome. Each covalent chemical modification of a protein leads to a new protein species. The primary synthesis product of translation represents a unique protein species, the initial protein species. This initial protein species may be proteolytically or environmentally processed, modified, and/or transported to organelles or outside the cell, spliced or degraded (Figure 4). For an individual cell the import of a specific protein species must also be considered. Additional protein species may result from nucleotide polymorphism such as different alleles, paralogs or alternative splicing sites. The term protein refers to its coding gene and therefore is the umbrella term for all of the developing protein species.

According to the nomenclature rules of IUBMB [26], the term "multiple forms of the enzyme" should be used as a broad term covering all proteins catalyzing the same reaction and occurring naturally in a single species and the term "isoenzyme" or "isozyme" should apply only to those multiple forms of enzymes arising from genetically determined differences in primary structure and not to those derived by modification of the same primary sequence. Here "isoenzymes" and extended "isoforms" are clearly genetically defined and exclude post-translational modifications. The reduction of multiple forms of an enzyme to a single species is at the end again a genetic definition. But, identical protein species of a certain enzyme isolated from mouse and human should have the same enzymatic properties. To avoid redundancies the pure chemical definition such as the protein species definition, independently of genetic origin (species, strain, individual, allel)) is a prerequisite for a unique terminology of functional proteomics.

Some of the most prominent examples for protein speciation are the histones. Alone for histone H3.2 over 150 different protein species were identified [27] after separation of the intact protein species. A histone code was postulated for different functions of different combinations of modifications [28]. Another example where it becomes obvious that the protein species and not the protein is the functional unit is tyrosine phosphatase 1B (PTP1B) [29]. PTP1B is converted into a sulphenyl-amide species at Cys 215. This oxidized protein species was identified by MALDI mass spectrometry. Oxidation causes large conformational change in the catalytic site that inhibits substrate binding. The oxidation to sulphenyl-amide represents a redox regulation of an enzyme. Other examples for function changes by post-translational modification are the angiotensin-converting enzyme [30,31] and GAPDH [32]. The speciation and function of the protein species arising from a given gene may be presented by the schema shown in figure 4.

The phenomenon of protein speciation is universal and not restricted to eukaryotes. In mycobacteria for example, in 2-DE patterns of cellular and supernatant proteins we identified 14 and 8 spots containing peptides of HspX [33] and Tuf [34], respectively. In the case of *Helicobacter pylori *we identified 647 spots containing proteins derived from 356 genes. The mean number of protein species derived from one gene was 2.08 and proteins derived from genes such as groEL, tufB and ureA occurred with 37, 23 and 18 protein species, respectively, on the 2-DE pattern (unpublished data). The real numbers of existing protein species are due to the limitations in detection and identification sensitivity for sure much higher. This is underpinned by the observation that speciation is even more extensively recognized in proteins with high abundance. It has to be considered that analogous to the uncertainty principle in quantum physics, present technology in proteomics may influence the protein species composition. Methylation, oxidation of methionines, tryptophans and cysteines, phosphorylations, amidations and deamidations may be caused by the method of preparation, separation, detection or identification. The native character of a modification has to be confirmed by a combination of several methods.

**Figure 5:**
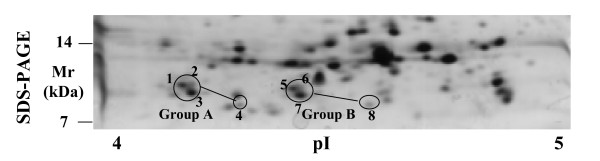
Cell culture supernatant proteins of *Mycobacterium tuberculosis *separated by a narrow range pH gradient between pI 4 and 5 in the first dimension and SDS-PAGE in the second dimension. Spots 1 to 8 were identified as different protein species of ESAT6 with 100% sequence coverage by MALDI-MS [35]. All spots of group A have an acetylated N-terminus. Spot 4 and 8 have a truncated C-terminus.

It becomes clear that the protein species level cannot be ignored when performing comprehensive proteome investigations. An investigation at the protein species level assumes identification with 100% sequence coverage. In mycobacteria the protein species derived from the gene of ESAT6 were found in 8 spots and 4 of these spots were identified with 100% sequence coverage. The modifications leading to speciation were C-terminal truncation and acetylation at the N-terminus [35] (Figure 5). Interestingly the acetylation of ESAT6 inhibits the interaction with another protein, CFP10. This interaction is important for the transfer of the virulence factor ESAT6 out of the bacterial cell into the host cell. In several cases a complete primary structure analysis is already possible by combination of different digestion procedures or using MS-based direct analysis of uncleaved proteins by the top-down approach [36]. One step towards the comprehensive analysis of protein species is the combination of top-down and bottom-up approaches [37-41]. Running et al. investigated ribosomes from the Gram-negative alpha-proteobacterium *Caulobacter crescentus*. They separated the proteins by a two-dimensional liquid chromatographic system that allowed the analysis of whole proteins by direct coupling to an ESI-QTOF mass spectrometer. In parallel the proteins of the fractions were enzymatically digested and analysed by a number of mass spectrometric methods. Ogorzalek Loo et al. [41] defined a combination of IEF of protein with MS of the intact proteins (top-down) with IEF of proteins or 2-DE with MS of peptides (bottom-up) as side-to-side proteomics. All of these approaches are time-consuming and technological developments are urgently needed to perform a proteome investigation at the protein species level in an acceptable time [42].

It is known that proteins with important functions such as ribosomal proteins have highly conserved sequences. Sequence databases contain already information on post-translational modification. In Uniprot this information is annotated for each sequence entry. A change of the basic unit protein sequence to protein species sequence including post-translational modifications should be considered. With the increase in proteomics data at the protein species level the conservation of post-translational modifications can now be investigated. This may help to validate the protein species identification and to elucidate a potential biological role.

### A new definition of the proteome

Avoidance of misleading protein expression terminology and awareness of the importance of post-translational modification means that the definition of the proteome has to be expanded by application of the protein species concept. The genome of an organism is with the exception of the rare event of mutation stable during the lifetime of an organism: The egg of a butterfly has the same genome as the butterfly itself. The protein species composition of an organism is however always changing during the development of an organism. The proteome of an organism is thus the sum of all of the protein species occurring during the lifetime of an organism. Here it becomes obvious that even more precision is necessary. The proteome has to be related to an individual. Following with line of thinking it becomes clear that the proteome of an organism cannot exist since each individual is living in different environment and produces an individual proteome. The term proteome has to be defined as:

### The proteome of an individual is defined by the sum and the time dynamics of all protein species occurring during the life-time of this individual

Determining the quantitative proteome of an individual would need measurements of the amount of each protein species from birth to death, a task far away from our current technological abilities. With the existing technologies we are able to investigate subproteomes at the organism level, ignoring the differences between individuals. The environmental and genetic influences are reduced as far as possible e.g. by the use of defined culture conditions, analyses at the same age or the use of inbred strains. These subproteomes can be defined in the following way:

### The subproteome is defined as the protein species composition of a biological compartment at a certain time and under defined environmental conditions

This definition considers the dependency of the proteome on environmental influences and the strong dynamic character of the proteome. For a more comprehensive analysis at the protein species level, time dependencies have to be included in a differential proteome analysis. Also, all other environmental factors have to be controlled as far as possible. To reach an understanding of biological processes, proteins have to be analyzed at the protein species level to cater for the influence of post-translational modifications on the function. For example, if we wish to gain information on activation of chromatin regions, which is caused by the acetylated protein species of histone 2a, it does not help to measure the concentration of histone 2a (the sum of all histone 2a protein species), but rather the amount of the acetylated H2a protein species has to be determined.

### Kinetic modeling of the proteome

As early as 1979, changes to protein patterns in response to stress or starvation stimuli were analyzed in *Escherichia coli *[43]. The field of physiological proteomics emerged from these investigations and was further developed to visualize protein concentrations depending on the time after a stimulus. Presenting a series of 2-DE gels from different time points after a stimulus results in some kind of film showing the dynamics of each of the protein species represented on the 2-DE pattern [44]. To reach the systems biology level, the influence of the time dimension is indispensable.

**Figure 6:**
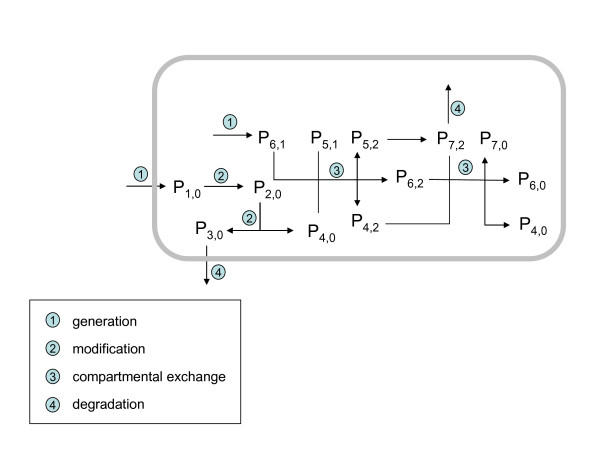
Illustration of the basic processes that may affect the dynamics of monomers. In this example, the monomer p_1,0 _is taken and transferred into the monomer p_2,0 _by chemical modification. p_2,0 _splits up into the monomers p_3,0 _and p_4,0 _(partial hydrolysis). p_4,0 _forms a trimer (compartment c = 3) with the dimer (compartment c = 1) constituted by the monomers 5 and 6. The trimer undergoes a chemical modification of its subunit 4 which is converted into the monomer 7. The trimer containing the monomers 4, 6 and 7 can be either degraded or decomposed into its monomeric subunits p_4,0_, p_6,0_, and p_7,0_.

In general, a protein species may undergo 4 different kinetic processes (Figure 6): (1) generation, including de novo synthesis as well as import into the system under study, (2) chemical modifications including attachment or detachment of functional groups, partial proteolysis and protein splicing, (3) exchange between compartments whereby binding to a macro-molecular structure (e.g. membrane) or encapsulation into an oligomeric protein complex such as the multimeric AMP-activated protein kinase [45] represents a specific form of compartmentation, (4) removal including complete proteolytic degradation and export from the system under study.

We denote an arbitrary protein species consisting of a single polypeptide chain (monomer) by p_i,c _where the index i = 1,..., NP counts the number of all possible monomeric protein species and the index c = 1,..., NC counts all possible protein compartments. By compartments we mean either a separate reaction space (e.g. cell organelle) and/or a macromolecular complex (e.g. ribosome) including the respective protein species.

The time-dependent change of the various protein species P_i,c _is described by the following set of kinetic equations:

(1) $$\frac{{\text{dp}}_{\text{i,c}}}{\text{dt}}={\text{g}}_{\text{i,c}}-{\text{d}}_{\text{i,c}}{\text{p}}_{\text{i,c}}+\sum_{\text{c'}=1}^{\text{NC}}\left[{\text{t}}_{\text{i,c:i,c'}}{\text{p}}_{\text{i,c'}}-{\text{t}}_{\text{i,c':i,c}}{\text{p}}_{\text{i,c}}\right]+\sum_{\text{i'}=1}^{\text{NP}}\left[{\text{q}}_{\text{i,c:i',c}}{\text{p}}_{\text{i',c}}-{\text{q}}_{\text{i',c:i,c}}{\text{p}}_{\text{i,c}}\right]$$

Here the 4 additive terms at the right-hand side refer to the following processes a protein species may undergo: generation (synthesis), degradation (proteolysis), chemical modification and compartmental exchange. The quantities g_i,c_, d_i,c_, t_i,c:i,c' _and q_i,c:i',c _denote the rate constants for these processes.

The first term on the right-hand side of equation (1) denotes the rate with which the protein species p_i,c _is produced per time unit. This rate depends on cellular mRNA levels and various regulatory events at transcriptional and post-transcriptional level so that in general g_i,c _will change over time.

The second term in equation (1) gives the degradation rate of p_i,c _As the molecular mechanisms determining the life span of proteins are still poorly understood the degradation is commonly treated as a first-order decay process, i.e. the degradation rate is proportional to the concentration of the protein species.

The sum in the third term of equation (1) covers all compartments c' which can communicate with compartment c. The first expression within the bracket refers to incoming processes, i.e. transitions of protein species p_i,c _from any compartment c' to compartment c, thereby increasing its concentration in c. The second expression in the bracket refers to outgoing processes, i.e. transitions of p_i,c _from compartment c to compartment c' thus decreasing its concentration in c.

The fourth term in equation (1) refers to all protein species that can be either derived from or converted into p_i,c _by chemical modification. Similar as in the sum of the third term, the two expressions in the bracket denote incoming and outgoing processes, i.e. increase of the concentration of p_i,c _due to chemical conversion p_i,c _→ p_i,c _or decrease of the concentration of p_i,c _due to chemical conversion p_i,c _→ p_i,c_

In the general equation system (1) many reaction rates are actually zero. For example, de novo synthesis of a protein species may take place either in the cytosol or the ER and generates the chemically non-modified form, i.e. the term g_i,c _is different from zero only for c = cytosol or c = ER and i referring to the non-modified species. Likewise, the transition rate t_i,c:i,c' _is different from zero only for adjacent compartments c and c', which are directly connected by a transport process.

Note that in the more simplistic case where compartmentalization and chemical modification of protein species are neglected equation (1) reduces to

(2) $$\frac{{\text{dp}}_{\text{i}}}{\text{dt}}={\text{g}}_{\text{i}}-{\text{d}}_{\text{i}}{\text{p}}_{\text{i}}$$

which for a sufficiently short time interval Δt = t - t_0 _is solved by

(3) p_i _≈ p_io _+ (g_i _-d_i_p_io_)Δt

with p_io _being the concentration of the species at time t_0_. Equation (3) has been originally proposed by Julka and Regnier [46]. Thus, equation (1) represents an extension of equation (2) to finite time scales under inclusion of compartmentalization and chemical conversions of protein species.

In a slightly more complex situation where any gene is associated with two protein species that are converted into each other by reversible phosphorylation of a single residue, the general equation (1) reduces to

(4) $$\begin{array}{l} {\text{p}}_{\text{x}}\approx {\text{p}}_{\text{xo}}+\left[\left({\text{g}}_{\text{x}}+{\text{t}}_{\text{x,y}}{\text{p}}_{\text{yo}}\right)-\left({\text{d}}_{\text{x}}+{\text{t}}_{\text{y,x}}{\text{p}}_{\text{xo}}\right)\right]\Delta \text{t}\\ {\text{p}}_{\text{y}}\approx {\text{p}}_{\text{yo}}+\left[\left({\text{g}}_{\text{y}}+{\text{t}}_{\text{y,x}}{\text{p}}_{\text{yo}}\right)-\left({\text{d}}_{\text{y}}+{\text{t}}_{\text{x,y}}{\text{p}}_{\text{yo}}\right)\right]\Delta \text{t} \end{array}$$

with p_x _and p_y _denoting here the non-phosphorylated and phosphorylated species, respectively. The transition rates t_y,x _and t_x,y _depend on the activities of the specific protein kinases and phosphatases involved in the chemical modification. Note that the two equations (4) are coupled: De-phosphorylation p_y _→ p_x _occurring during time span Δt appears as an additional generation process of p_x _and additional degradation process of p_y_. Likewise, phosphorylation p_x _→ p_y _appears as an additional generation process of p_y _and additional degradation process of p_x_. As long as the phosphorylation does not occur co-translationally, g_y _will be zero so that the generation term in the second equation for p_y _will be given by the phosphorylation rate t_y,x _p_yo_.

### Systems biology

Systems biology attempts to integrate data from diverse high-throughput technologies, such as genomics, transcriptomics, proteomics and metabolomics using bioinformatics. Even with the high sensitivity of today's proteomics methods, proteome coverage is low compared to microarray technology. The reasons could be that not all of the transcribed genes are translated at a certain time point or that the dynamic range of protein amount reaching up to 12 and more orders of magnitude is not covered by the currently available proteomic technologies. The low correlation between DNA microarray and proteomics data [47] has also implicated extensive molecular control at the level of translation and post-translational modification. These discrepancies are caused by the factors which influence the dynamics at the proteomic level [46]. We have developed a more exact terminology which will help us to understand the dynamics of proteomics in more detail. If one accepts the protein species as the functional unit of the proteome, it becomes clear that it is impossible to assume a simple, linear relationship between the level of an mRNA and the amount of its encoded protein or even one of its encoded protein species. For the understanding of a cell or an organelle genomics, transcriptomics and proteomics complement each other.

Proteomics has to consider a series of parameters, which have to be clearly separated for systems biology. Here already the sample preparation decides which part of the proteome will be covered. Prefractionation into different cell compartments or protein complexes and solubilization decide which protein classes are investigated. Deregulating the system by physical or chemical parameters, e.g. by influencing the protein species composition by temperature or by a drug has to be performed under controlled conditions ensuring that no other parameters are influencing the system. A main limitation for a clear definition of the sample, which is analysed, is the purity of it. An eye lens can be prepared without substantial impurities, whereas a certain area of the brain is difficult to prepare without contamination of surrounding areas. Quantification may be achieved by optical density measurements on 2-DE gels or by MS peak area determination using label-free quantification [48]. Parallel quantification in one gel or one LC run is supported by fluorescence [49] or isotope labelling [50]. For quantification only the procedures separating the protein species before digestion have the advantage that the quantity of the protein species can be determined.

Since classical proteomic approaches (excluding shot-gun or bottom-up approaches) alone provide mainly information on the relative *amount *of protein species and only in certain cases information about the *activity *of these protein species, it is necessary to complement classical proteomic approaches by metabolomics and interaction studies to reach the functional level of the biological system under investigation. Surely the attempt of a clear terminology in proteomics will help to contribute to the deciphering of biological systems.

## Conclusion

Proteomics developed very fast within the last 15 years and large scale investigations at the protein level are possible. Principally a complete structure analysis of each protein species separated is already possible. Today the challenge in proteomics is to reach 100% sequence coverage and identification of all post-translational modifications within each protein species in higher throughput. Protein speciation has to be realized experimentally and for theoretical considerations. This will clearly improve data mining to understand biological phenomena based on proteomic investigations.

## Authors' contributions

Peter R. Jungblut has developed the concept, has written the text, and contributed with Figures 1 to 5. H.G. Holzhütter has contributed with the kinetic modeling and Figure 6.

R. Appweiler has revised critically the text. H. Schlüter contributed to the concept and revised critically the text.
